# Conditioned medium derived from FGF-2-modified GMSCs enhances migration and angiogenesis of human umbilical vein endothelial cells

**DOI:** 10.1186/s13287-020-1584-3

**Published:** 2020-02-18

**Authors:** Shanshan Jin, Chengzhe Yang, Jiahui Huang, Lianlian Liu, Yu Zhang, Shutong Li, Liguo Zhang, Qinfeng Sun, Pishan Yang

**Affiliations:** 1 Department of Periodontology, School and Hospital of Stomatology, Shandong University, No.44-1 Wenhua Road West, Jinan, 250012 Shandong China; 2 Shandong Provincial Key Laboratory of Oral Tissue Regeneration, Jinan, Shandong China; 3 Shandong Engineering Laboratory for Dental Materials and Oral Tissue Regeneration, Jinan, Shandong China; 4 Department of Stomatology, Qilu Hospital of Shandong University, Jinan, Shandong China

**Keywords:** Conditioned medium, FGF-2, Angiogenesis, hGMSCs, HUVECs, Overexpression

## Abstract

**Background:**

Angiogenesis plays an important role in tissue repair and regeneration, and conditioned medium (CM) derived from mesenchymal stem cells (MSC-CM) possesses pro-angiogenesis. Nevertheless, the profile and concentration of growth factors in MSC-CM remain to be optimized. Fibroblast growth factor-2 (FGF-2) has been proven to be an effective angiogenic factor. Thus, the aim of this study was to verify whether FGF-2 gene overexpression optimized CM from human gingival mesenchymal stem cells (hGMSCs) and whether such optimized CM possessed more favorable pro-angiogenesis effect.

**Methods:**

First, FGF-2 gene-modified hGMSCs were constructed using lentiviral transfection technology (LV-FGF-2^+^-hGMSCs) and the concentration of angiogenesis-related factors in LV-FGF-2^+^-hGMSC-CM was determined by ELISA. Then, human umbilical vein endothelial cells (HUVECs) were co-cultured for 3 days with LV-FGF-2^+^-hGMSC-CM, and the expression level of placenta growth factor (PLGF), stem cell factor (SCF), vascular endothelial growth factor receptor 2 (VEGFR2) in HUVECs were determined by qRT-PCR, western blot, and cellular immunofluorescence techniques. The migration assay using transwell and in vitro tube formation experiments on matrigel matrix was conducted to determine the chemotaxis and angiogenesis enhanced by LV-FGF-2^+^-hGMSC-CM. Finally, NOD-SCID mice were injected with matrigel mixed LV-FGF-2^+^-hGMSC-CM, and the plug sections were analyzed by immunohistochemistry staining with anti-human CD31 antibody.

**Results:**

LV-FGF-2^+^-hGMSC-CM contained significantly more FGF-2, vascular endothelial growth factor A (VEGF-A), and transforming growth factor β (TGF-β) than hGMSC-CM. HUVECs pretreated with LV-FGF-2^+^-hGMSC-CM expressed significantly more PLGF, SCF, and VEGFR2 at gene and protein level than hGMSC-CM pretreated HUVECs. Compared with hGMSC-CM, LV-FGF-2^+^-hGMSC-CM presented significantly stronger chemotaxis to HUVECs and significantly strengthened HUVECs mediated in vitro tube formation ability. In vivo, LV-FGF-2^+^-hGMSC-CM also possessed stronger promoting angiogenesis ability than hGMSC-CM.

**Conclusions:**

Overexpression of FGF-2 gene promotes hGMSCs paracrine of angiogenesis-related growth factors, thereby obtaining an optimized conditioned medium for angiogenesis promotion.

## Background

Periodontitis, bone fractures, malformations, and surgical removal of tumors can cause oral and maxillofacial bone defects and interfere with normal function and configuration. Regeneration of these damaged bone tissue has become an important clinical problem [1]. Currently, bone grafts, including autografts, allografts, xenografts, and synthetic grafts, are the primary treatment modalities [2, 3]. However, these bone grafts are limited in clinical application for varied reasons. Constraints of autografts lie in availability, prolonged operation time and accompanied donor site morbidity, while allografts bring patients at risk for infections and immune rejection [4, 5]. The main drawback of xenografts and synthetic grafts is that they do not elicit any osteoinductive or osteogenic potential on their own [6]. The new options are needed to improve bone regeneration outcomes.

Mesenchymal stem cells (MSCs) offer a potential strategy for tissue repair and wound healing. MSCs are multipotent somatic stem cells, which can be obtained from a number of adult tissues including the bone marrow, adipose tissue [7], periodontal ligament [8, 9], dental pulp [10, 11], and gingivae [12, 13]. MSCs have been used to treat a wide range of diseases, including bone defect regeneration [14]. However, MSCs transplantation has some limitations impeding its clinical use [15, 16], such as the poor engraftment, a relatively short life span and potential tumorigenesis of transplanted MSCs.

Recently, it has been discovered that, in addition to their direct differentiation into tissue-related cell type, MSCs behave a potential paracrine effect, which includes anti-inflammatory and/or immunomodulation; anti-apoptosis; angiogenesis, stimulating the growth and differentiation of surrounding cells [17]; and chemoattraction properties [18, 19]. Moreover, some scholars even argued that instead of directly transforming into a tissue-related cell type, transplanted MSCs promote the proliferation and differentiation of autologous MSCs by regulating the microenvironment of the lesion site [20, 21]. Therefore, the regenerative potential of MSC therapies has been found—at least in part—to be mediated via such paracrine actions. Furthermore, transplantation of conditioned medium (CM) containing paracrine factors such as the insulin-like growth factor (IGF-1) and vascular endothelial growth factor (VEGF) has been reported to enhance wound healing and bone regeneration in animal models [22–24].

Although MSC-CM therapy appears to be a highly promising alternative to MSC transplantation, a major constrain in its clinical application is that the concentrations of growth factors in CM are too low for therapeutic use [20]. For that, some scholars manage to optimize the profile and concentration of growth factors and cytokines in MSC-CM by preconditioning MSCs with inflammatory stimuli [21] or growth factors [22].

There is growing evidence that tissue repair and regeneration are closely related to vascularization. Vascular endothelial growth factor A (VEGF-A), one of the most important regulators of angiogenesis [23] plays a crucial role during endochondral ossification. In VEGF-A conditional knockout (CKO) mice, the lack of VEGF-A in chondrocytes leads to impaired embryonic bone development, reduced removal of terminally differentiated hypertrophic chondrocytes, and death of a large number of cells in the femur. This evidence demonstrates the important role of angiogenesis during skeletal development [24]. The combined use of stem cells and endothelial cells has a stronger effect on repairing cardiac damage than using stem cells or endothelial cells alone [25]. Recent studies have shown that the improved vascularization strategies in vitro can greatly enhance the regeneration of bone tissue in vivo [26]. These backgrounds suggest that pro-angiogenesis is an important strategy for promoting tissue regeneration.

Numerous studies have confirmed that FGF-2 produced by mesoderm and neuroectodermal cells acts as an effective angiogenic factor to promote microvascular formation [27]. The ERK1/2-MAP kinase signaling pathway controls the proliferation of various cell types. Studies have shown that FGF-2 can promote the phosphorylation of ERK1/2 and thereby promote endothelial cell proliferation [28]. FGF-2 may increase the secretion of VEGF by vascular smooth muscle endothelial cells and Muller cells through the activation of PI3K, which promotes angiogenesis [29]. Moreover, FGF-2 has been proven to stimulate bone marrow mesenchymal stem cell (BMMSC) proliferation and maintain its multipotent while being added to the culture medium [30]. Our previous studies have also found that preconditioning with FGF-2 could enhance sustained proliferation and osteogenic differentiation capacity of stem cells from periodontal ligaments (PLDSCs) [31, 32]. This implies that FGF-2 possesses a potential effect on angiogenesis and osteogenesis, and preconditioning with FGF-2 is one of the effective approaches to optimize MSC-CM.

Given the potential effect of FGF-2 on angiogenesis, osteogenesis, anti-inflammatory, immunoregulatory, and osteogenic effect of MSC-derived from gingivae (GMSCs), it is speculated that conditioned medium from FGF-2 overexpressed GMSCs may possess better angiogenesis and osteogenesis ability. Thus, in this study, FGF-2 gene-modified GMSCs (FGF-2^+^-GMSCs) were constructed, angiogenesis-associated cytokines in CM from FGF-2^+^-GMSCs were detected, and we used lentivirus as a vector to increase the concentration of FGF-2 of the conditioned medium by overexpressing the FGF-2 gene and increasing the amount of FGF-2 secreted by the gingival mesenchymal stem cells, and hence promote the role of blood vessel formation.

## Materials and methods

### Human GMSCs culture and identification

The program adhered to guidelines of patients’ consent for participation and research was supported by the Ethics Committee of Stomatological Hospital, Shandong University (No. 201712). Healthy human gingival tissues were obtained from 6 patients in the Stomatological Hospital of Shandong University and cultured in Dulbecco’s modified Eagle’s medium (DMEM, Hyclone, Logan, UT, USA) which contained 5% antibiotics (100 U/ml penicillin G, 10 mg/ml streptomycin). After washed five times with phosphate-buffered solution (PBS, Hyclone, Logan, UT, USA) and cut into small pieces about 1 mm^2^ within 2 h, the tissues were seeded at the bottom of 25 cm^2^ cell culture flask (Corning, NY, USA) and cultured in DMEM with 20% fetal bovine serum (FBS, BioInd, Kibbutz, Israel) and 1% antibiotics at 37 °C in a 5% CO^2^ incubator. The primary cells were purified by limiting-dilution to obtain stem cells. Then, the stem cells were cultured in a 10-cm cell-cultured dish in DMEM containing 10% FBS. Cells were passaged with 0.25% trypsin/EDTA solution (Hyclone, Logan, UT, USA) when reached 80–90% confluency, and cells were used in the following experiments at passage 3. To identify the characteristics of GMSCs, colony formation unit, cell surface antigens, and in vitro osteogenic and adipogenic assays were performed as previously reported [12].

### LV-FGF-2^+^-hGMSC construction

The lentivirus vector construction carrying the FGF-2 (pHBLV-CMV-MCS-3FLAG-EF1-ZsGreen-T2A-PURO-FGF-2) or control vector (pHBLV-CMV-MCS-3FLAG-EF1-ZsGreen-T2A-PURO) and lentiviral generation were performed by the Hanbio Biotechnology Co., Ltd. (Shanghai, China). To construct LV-FGF-2^+^-hGMSCs, 2 × 10^5^ hGMSCs suspended in DMEM were plated into a 6-well plate. After 24 h, cells were then incubated with the corresponding lentiviral particles (MOI = 40) in 1 ml of the regular culture medium supplemented with 6 μg/ml polybrene. Four hours later, another 1 ml of the regular culture medium was added to each well. The cells were incubated at 37 °C for 24 h before the medium was switched to a regular culture medium. After 48 h, green fluorescence was captured by a fluorescence microscope (Olympus, Tokyo, Japan). Stably transduced cells were selected with 1 μg/ml puromycin (Sigma, Louis, MO) and maintained in culture medium containing 0.5 μg/ml puromycin.

### Preparation of CM

hGMSCs, LV-vector^+^-hGMSCs, and LV-FGF-2^+^-hGMSCs (1 × 10^6^ cell/dish) were seeded in 10-cm cell-cultured dishes separately and cultured in DMEM with 10% FBS. At 80% confluence, the cells were washed three times with PBS and the media were replaced with a 10-ml serum-free DMEM. After 3 days, the CM was centrifuged at 1000 rpm for 10 min, filtered with 0.22 μm filter, and then concentrated using a 10-kDa ultrafiltration centrifuge tube (Millipore, USA) at 8000 g of centrifugal force at 4 °C for 1 h. The conditioned mediums were concentrated approximately 50 times. The collected CM was stored in a − 80 °C refrigerator for subsequent experiments.

### ELISA

The relative expression levels of VEGF-A, TGF-β, and FGF-2 in CM were analyzed by using ELISA kits (Dakewe, Beijing, China), and the absorbance values were detected at 450 nm wavelength.

### HUVECs culture and pretreatment

Human umbilical vein endothelial cells (HUVECs; ScienCell, USA) were cultured in endothelial cell medium (ECM; ScienCell, USA) with 5% fetal bovine serum (FBS; ScienCell, USA), 1% endothelial cell growth supplement (ECGS; ScienCell, USA), and 1% penicillin/streptomycin solution (P/S; ScienCell, USA) in 37 °C incubator with 5% CO^2^ and 95% air. HUVECs were passaged when the cells reached 80–90% confluency. Cells were used for subsequent experiments at passages 4–7. The HUVECs were preconditioned by LV-FGF-2^+^-hGMSC-CM, hGMSC-CM, or LV-vector^+^-hGMSC-CM for 3 days, and ECM (ScienCell, USA) without CM was chosen as the negative control. Then, the cells were cultured by ECM with 5% fetal bovine serum (FBS; ScienCell, USA), 1% endothelial cell growth supplement (ECGS; ScienCell, USA), and 1% penicillin/streptomycin solution (P/S, ScienCell) for 7 to 10 days.

### Quantitative real-time polymerase chain reaction (qRT-PCR)

To identify the transfection efficiency by lentivirus, human GMSCs (2 × 10^6^ cell/well) transfected by lentivirus carrying the FGF-2 gene or control vector were seeded in 6-well plates containing DMEM with 10% FBS. When the cells were fused to 80–90%, total RNA of hGMSCs were isolated by RNAiso Plus (Takara, Kusatsu, Japan), extracted with chloroform-isopropanol, and precipitated with ethanol. After being quantified, the complementary DNA (cDNA) was reverse transcribed by using PrimeScript™ RT reagent Kit and DNA Eraser (Takara). qRT-PCR quantitative assay was performed with LightCycler 96 Real-Time PCR System (Roche, Basel, Switzerland) by using TB GREEN™ Premix Ex Taq™ II (Takara). The primer sequences and housekeeping gene GAPDH were listed in Table 1.

**Table 1:** Primer sequences for qRT-PCR

Gene	Forward (5′-3′)	Reverse (5′-3′)
GAPDH	GCACCGTCAAGGCTGAGAAC	TGGTGAAGACGCCAGTGGA
FGF-2	GAGCGACCCTCACATCAA	CGTTTCAGTGCCACATACC
PLGF	TTGTCTGCTGGGAACGGCTCGT	CCGGCACACAGTGCAGATTCT
SCF	GACCTTGTGGAGTGCGTGAA	CTGGGTTCTGGGCTCTTGAAT
VEGFR2	CAAGTGGCTAAGGGCATGGA	ATTTCAAAGGGAGGCGAGCA

In order to identify the expression of angiogenesis-related genes, HUVECs pretreated with hGMSC-CM, LV-vector^+^-hGMSC-CM, or LV-FGF-2^+^-hGMSC-CM were cultured for another 7 to 10 days. Then, total RNA of HUVECs were isolated by RNAiso Plus (Takara, Kusatsu, Japan), extracted with chloroform-isopropanol, and precipitated with ethanol. After being quantified, the complementary DNA (cDNA) was reverse transcribed using PrimeScript™ RT reagent Kit and DNA Eraser (Takara). qRT-PCR quantitative assay was performed with LightCycler 96 Real-Time PCR System (Roche, Basel, Switzerland) using TB GREEN™ Premix Ex Taq™ II (Takara). The primer sequences and housekeeping gene GAPDH were listed in Table 1.

### Western blot analysis of angiogenesis-related proteins

The pretreated HUVECs were washed by pre-cooling PBS three times, and the proteins were extracted by RIPA lysis containing 1% PMSF (Solarbio, Beijing, China). The concentration of proteins was measured according to the BCA protein assay kit (Solarbio, Beijing, China). The proteins were boiled for 5 min in SDS-PAGE loading buffer, and 30 μg of proteins was loaded to each lane and separated by 12% SDS-PAGE gels and transferred onto polyvinylidene fluoride (PVDF; Millipore, Billerica, MA, USA) membranes. The membranes were blocked by 5% non-fat milk for 1 h and then immersed in primary antibodies overnight at 4 °C; the primary antibodies are as follows: rabbit anti-GAPDH (1:10000; Proteintech, Chicago, IN, USA), rabbit anti-PLGF (1:1000; Abcam, Cambridge, UK), rabbit anti-SCF (1:10000; Abcam), and rabbit anti-VEGFR2 (1:1000; Cell Signaling Technology, USA). The membranes were incubated with horseradish peroxidase-conjugated secondary antibodies (1:10000; Proteintech) for 1 h. Chemiluminescence reagents (Millipore) were used for the development of band visualization. ImageJ software was used to analyzed the protein expression levels.

### Immunocytochemistry

HUVECs were pretreated with hGMSC-CM, LV-vector^+^-hGMSC-CM, or LV-FGF-2^+^-hGMSC-CM for 3 days. HUVECs were fixed with 4% paraformaldehyde (BioSharp) for 15 min, then permeabilized the cells by 0.2% Triton-X-100 (Dingguo, Beijing, China) for 15 min. After being blocked with 10% goat serum for 1 h, cells were incubated with rabbit anti-VEGFR2 primary antibody (1:400; Cell Signaling) overnight. After washed three times with PBS, cells were incubated with goat anti-rabbit IgG secondary antibody (1:500; Proteintech) for 1 h at 37 °C. 2-(4-amidinophenyl)-6-indolecarba midine dihydrochloride (DAPI; Proteintech) was used to stain nuclei. Images were captured with a fluorescence microscope (OLYMPUS IX73, Tokyo, Japan) in the darkroom.

### Cell migration assay

To detect the effect of LV-FGF-2^+^-hGMSC-CM on migration ability of HUVECs, 8 × 10^4^ HUVECs were seeded at the upper chamber of the transwell chambers with 8-μm pores (Corning, NY, USA) containing 200 μl ECM with 0.1% FBS, and the lower chamber was injected with 500 μl ECM containing 0.1% FBS, 0.1% FBS + 10 μl hGMSC-CM, 0.1% FBS + 10 μl LV-vector^+^-hGMSC-CM, 0.1% FBS + 10 μl LV-FGF-2^+^-hGMSC-CM, or 5% FBS, respectively. After incubating at 37 °C for 20 h, the HUVECs on the upper surface of the upper chamber were cleaned with cotton swabs and the HUVECs on the lower surface of the upper chamber were fixed with 4% paraformaldehyde (Biosharp, China) for 15 min and then stained with 0.1% crystal violet solution (Sigma-Aldrich) for 10 min. The stained cells were observed under an inverted microscope (Olympus, Tokyo, Japan) at × 400 magnification.

### Tube formation assay

To visually analyze the angiogenic ability of LV-FGF-2^+^-hGMSC-CM, a 60-μl matrigel matrix (Corning) was transferred to a 96-well plate and then incubated in 37 °C incubator for 30 min. The HUVECs (3 × 10^4^ cells/well) were seeded on the matrigel matrix with 400 μl ECM containing 5% FBS, 5% FBS + 8 μl hGMSC-CM, 5% FBS + 8 μl LV-vector^+^-hGMSC-CM, or 5% FBS + 8 μl LV-FGF-2^+^-hGMSC-CM, respectively, and incubated at 37 °C for 4 to 24 h. The tube-like structures were photographed under an inverted microscope (Olympus, Tokyo, Japan) at × 40 magnification, and the total tube length and total branching length were analyzed by Image J software.

### Animal experiment and immunohistochemistry staining

NOD-SCID mice (6 to 8 weeks of age) from Beijing Vital River Laboratory Animal Technology Co., Ltd. (Beijing, China) were used to detect the ability of LV-FGF-2^+^-hGMSC-CM in promoting HUVEC angiogenesis in vivo. These NOD-SCID mice were assigned to four groups: matrigel group (500 μl matrigel), HUVEC group (500 μl matrigel + 5 × 10^6^ HUVECs), hGMSC-CM group (500 μl matrigel + 5 × 10^6^HUVECs + 100 μl hGMSCs-CM), and LV-FGF-2^+^-hGMSC-CM group (500 μl matrigel + 5 × 10^6^ HUVECs + 100 μl LV-FGF-2^+^-hGMSC-CM). Matrigel or matrigel mixture was injected subcutaneously into the lower dorsal region of SCID mice. After 7 days, the matrigel plugs were removed, fixed in 4% paraformaldehyde for 24 h, rinsed in running water for 4 h, dehydrated, embedded in paraffin blocks, and sectioned into 5 mm. Then, immunohistochemistry staining was performed according to the protocol of the immunohistochemical stain kit (ZCGB-BIO, Beijing, China). Briefly, after dewaxing and dehydration, the sections were immersed in primary anti-human CD31 antibody (1: 200; Bioss, Beijing, China) overnight and immersed in biotin secondary antibody for 30 min. After staining with DAB stain solution (ZCGB-BIO, Beijing, China), the sections were counter-stained by using hematoxylin for 3 min and eosin for 20 s. Images were captured under an inverted microscope (Olympus, Tokyo, Japan) at × 200 and × 400 magnification.

### Statistical analysis

SPSS 19.0 was used to conduct all the statistical analyses. Data were expressed as mean ± standard error of the mean (S. E. M) of three independent experiments. Differences among groups were evaluated by one-way ANOVA followed by Tukey HSD comparison test. *P* values less than 0.05 were considered to be statistically significant.

## Results

### hGMSCs culture and identification

**Fig. 1:**
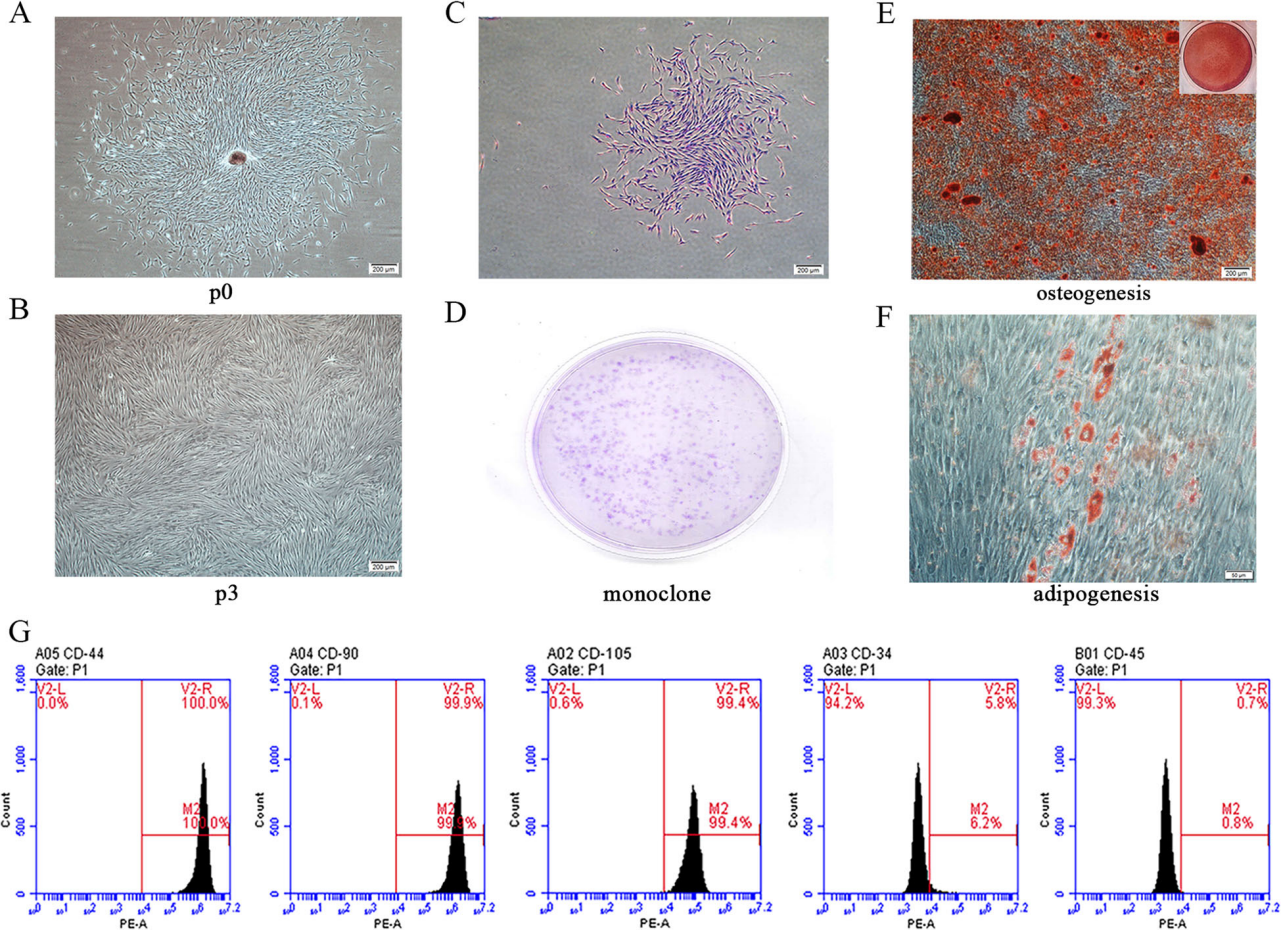
Human GMSCs culture and identification. Human GMSCs were obtained by tissue block digestion-limited dilution method, and the third-generation hGMSCs were used for subsequent experiments (**a**, **b**). In the flow cytometric analysis, hGMSCs positively expressed mesenchymal stem cell surface markers CD44 (100%), CD90 (99.9%), and CD105 (99.4%), but negatively expressed hematopoietic stem cell surface markers CD34 (5.8%) and CD45 (0.7%). Negative control single peak is located on the left side of the vertical line (**g**). The clones were obtained by hGMSCs culturing for 12 days at 500 cells per dish (**c**, **d**). After 21 days of osteogenic induction, hGMSCs formed mineralized nodules that stained with alizarin red (**e**). After 21 days of adipogenic induction, hGMSCs formed lipid droplets stained by oil red O (**f**)

The hGMSCs were obtained by tissue block digestion-limited dilution method (Fig. 1a, b). To identify hGMSCs, the cell surface antigens were analyzed by flow cytometry; the positive indicators were CD44 (100%), CD90 (99.9%), and CD105 (99.4%), and the negative indicators were CD34 (5.8%) and CD45 (0.7%) (Fig. 1g). hGMSCs have the potential of osteogenic differentiation and adipogenic differentiation (Fig. 1e, f). The colony-forming unit assay demonstrated that the human gingival mesenchymal stem cells have the ability to form clones (Fig. 1c, d).

### LV-FGF-2 transfection promotes FGF-2 gene expression and FGF-2, VEGF-A, and TGF-β paracrine of hGMSCs

**Fig. 2:**
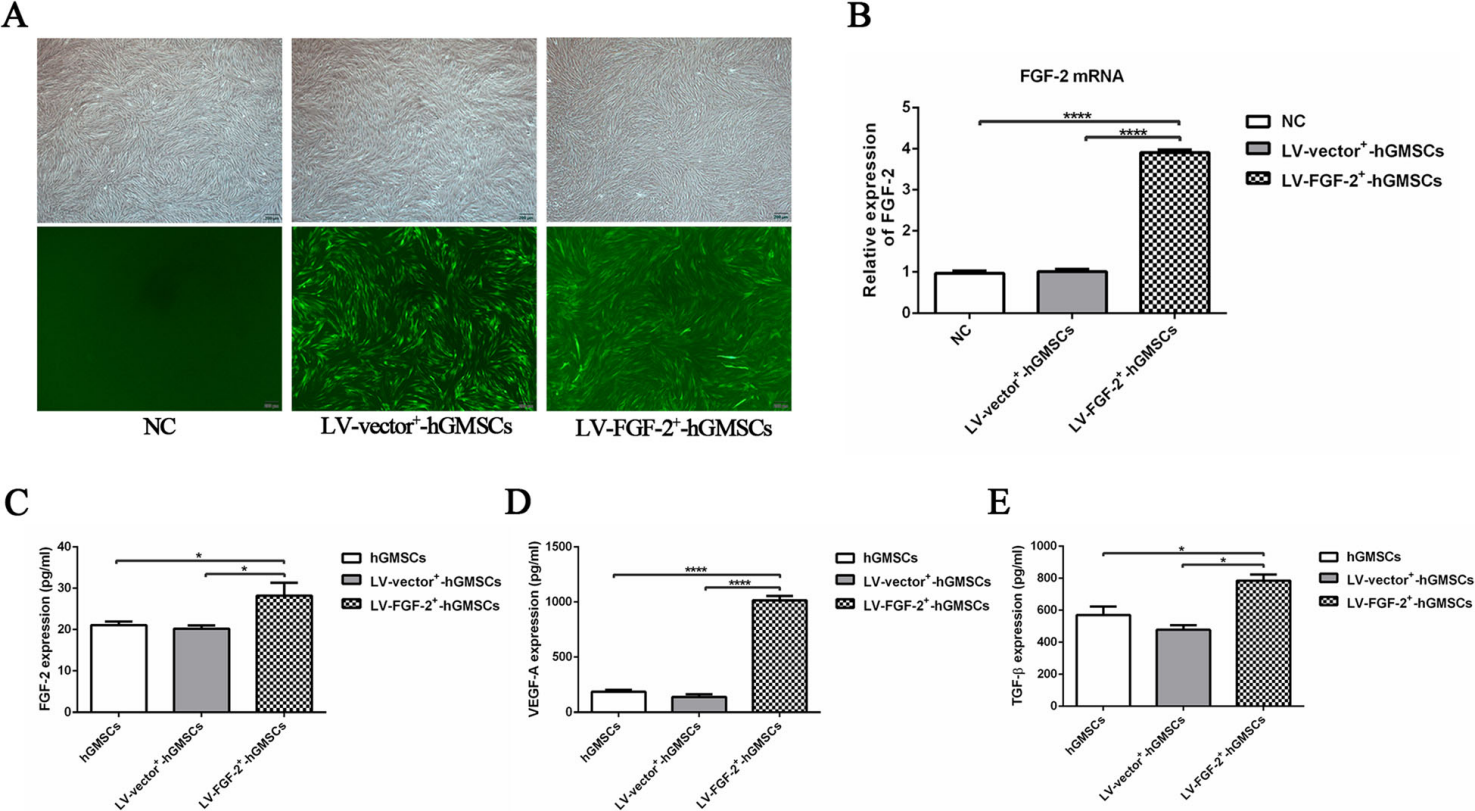
The lentiviral transfection efficiency and its effect on the paracrine of hGMSCs. Human GMSCs transfected with LV-vector^+^ or LV-FGF-2^+^ (MOI = 40) were observed under an inverted microscope (**a**). The lentiviral transfection efficiency was detected by qRT-PCR (**b**). The transfected cells were cultured by free-serum medium for 3 days when they converged to 80–90%. Angiogenesis-related factors FGF-2, VEGF-A, and TGF-β in the supernatant were detected by ELISA. The concentration of FGF-2, VEGF-A, and TGF-β in LV-FGF-2^+^-hGMSC-CM is higher than other two groups; however, there was no statistical difference between hGMSC-CM and LV-vector^+^-hGMSC-CM (**c**–**e**). *p < 0.05, ****p < 0.0001

The lentivirus vector pHBLV-CMV-MCS-3FLAG-EF1-ZsGreen-T2A-PURO-FGF-2 was successfully constructed as shown by the map of the plasmid (Additional file 1). The green fluorescence staining showed that the lentivirus was high in the hGMSCs when MOI = 40 (Fig. 2a). The qRT-PCR indicated that LV-FGF-2^+^-hGMSCs expressed higher FGF-2 compared with the LV-vector^+^-hGMSCs (Fig. 2b). ELISA assay showed that the concentrations of FGF-2 (Fig. 2c), VEGF-A (Fig. 2d), and TGF-β (Fig. 2e) in LV-FGF-2^+^-hGMSC group significantly increased compared to those in LV-vector^+^-hGMSC group and hGMSC group.

### LV-FGF-2^+^-hGMSC-CM preconditioning enhances the expression of angiogenesis-related factors in HUVECs

**Fig. 3:**
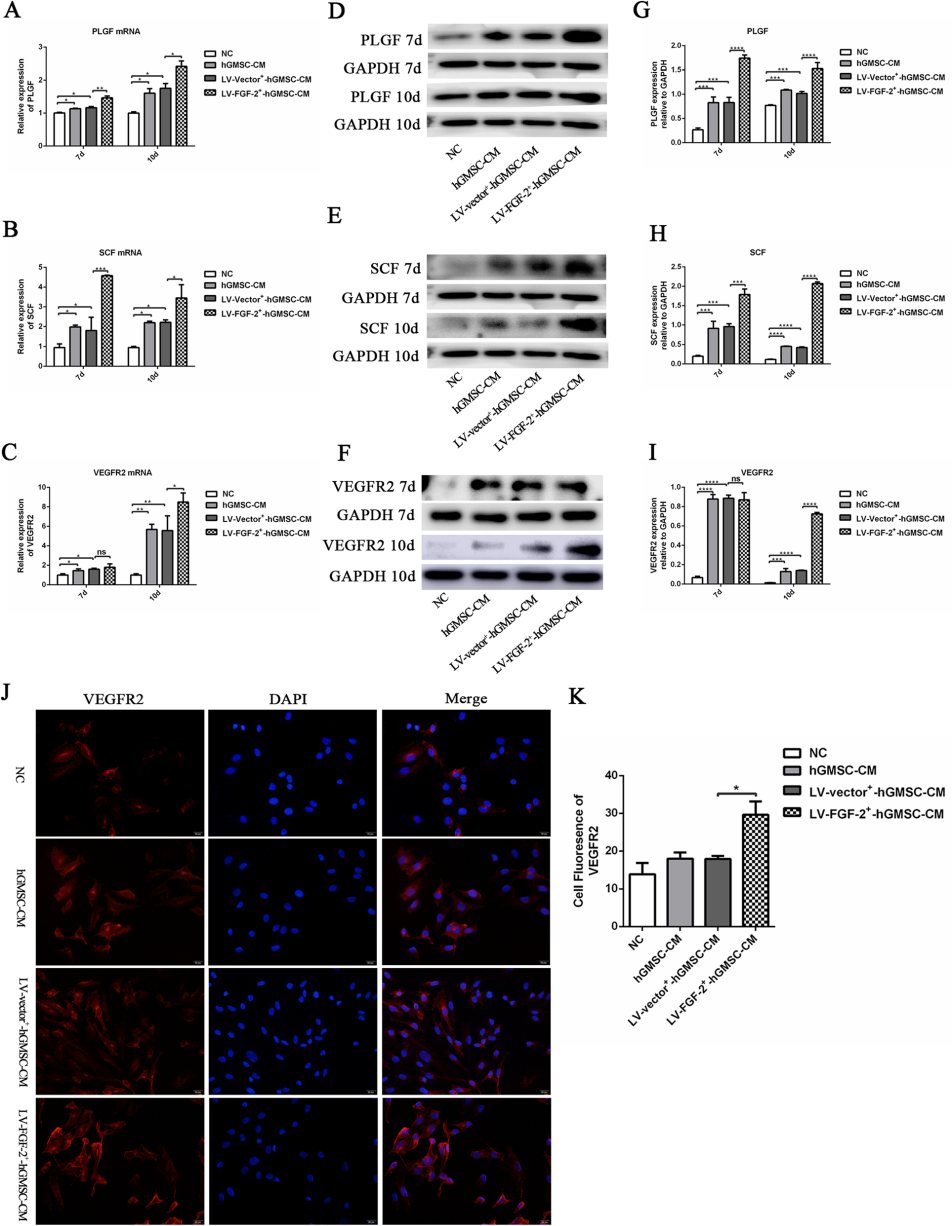
The effect of LV-FGF-2^+^-hGMSC-CM on the expression of angiogenesis-related factors in HUVECs. HUVECs were pretreated with hGMSC-CM, LV-vector^+^-hGMSC-CM, or LV-FGF-2^+^-hGMSC-CM for 3 days (negative control group was cultured by ECM) and then re-cultured by ECM for 7 to 10 days. The mRNA and protein expressions of PLGF, SCF, and VEGFR2 were determined by qRT-PCR (**a**–**c**) and western blot (**d**–**i**). The VEGFR2 protein expression was also detected by immunofluorescence staining (VEGFR2, red fluorescent signals; DAPI, blue signals; × 400 magnification) (**j**, **k**). ns = no significant difference, *p < 0.05, **p < 0.01, ***p < 0.001, ****p < 0.0001

SCF, PLGF, and VEGFR2 play significant roles in angiogenesis; thus, their expression in HUVECs undergoing different preconditioning treatments was assayed to reflect the angiogenesis potential of LV-FGF-2^+^-hGMSC-CM. As shown in Fig. 3, PLGF (Fig. 3a, d, g), SCF (Fig. 3b, e, h), and VEGFR2 (Fig. 3c, f, i) mRNA and protein expressions in HUVECs preconditioned by LV-FGF-2^+^-hGMSC-CM were significantly higher (except for VEGFR2 protein expression at 7th day) than in those preconditioned by LV-vector^+^-hGMSC-CM, hGMSC-CM, or negative control group. And PLGF, SCF, or VEGFR2 mRNA and protein expressions in the LV-vector^+^-hGMSC-CM group and hGMSC-CM group were also significantly higher than those in the negative control group (Fig. 3a–i).

Immunofluorescence staining presented a similar result. As shown in Fig. 3j and Fig. 3k, the VEGFR2 expression in HUVECs pretreated by LV-FGF-2^+^-hGMSC-CM was significantly higher than in those pretreated by hGMSC-CM and LV-vector^+^-hGMSC-CM. LV-vector^+^-hGMSC-CM group and hGMSC-CM group also had a tendency to increase VEGFR2 expression, but no significant difference was arrived.

### LV-FGF-2^+^-hGMSC-CM promotes migration of HUVECs

**Fig. 4:**
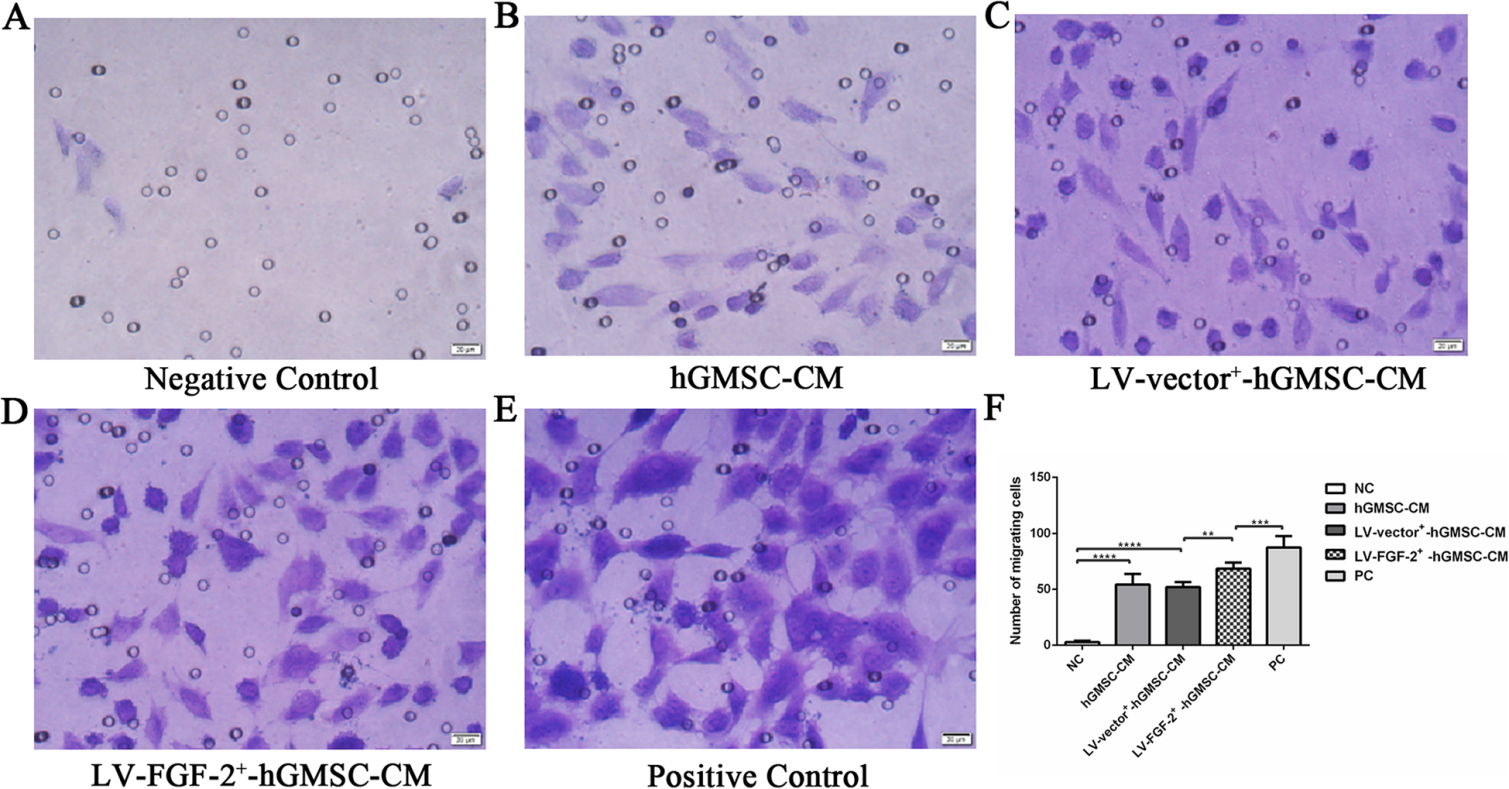
The effect of LV-FGF-2^+^-hGMSC-CM on the migration of HUVECs. HUVECs were cultured in the upper chamber of the transwell chambers with 0.1% FBS, and 0.1% FBS (NC), 0.1% FBS + hGMSC-CM, 0.1% FBS + LV-vector^+^-hGMSC-CM, 0.1% FBS + LV-FGF-2^+^-hGMSC-CM, or 5% FBS (PC) was injected in the lower chamber. After 20 h, the number of cells on the lower surface of the upper chamber was counted under an inverted microscope at × 400 magnification (**a**–**e**) and statistically analyzed among five groups (**f**). **p < 0.01, ***p < 0.001, ****p < 0.0001

The migration assay by using transwell chambers revealed that LV-FGF-2^+^-hGMSC-CM significantly increased the number of migrating HUVECs compared with ordinary medium, hGMSC-CM, or LV-vector^+^-hGMSC-CM. And the numbers of migrating HUVECs in the hGMSC-CM group and LV-vector^+^-hGMSC-CM group were significantly more than those in the negative control group. As expected, the positive control group had the strongest promoting effect (Fig. 4a–f).

### LV-FGF-2^+^-hGMSC-CM increases tube formation

**Fig. 5:**
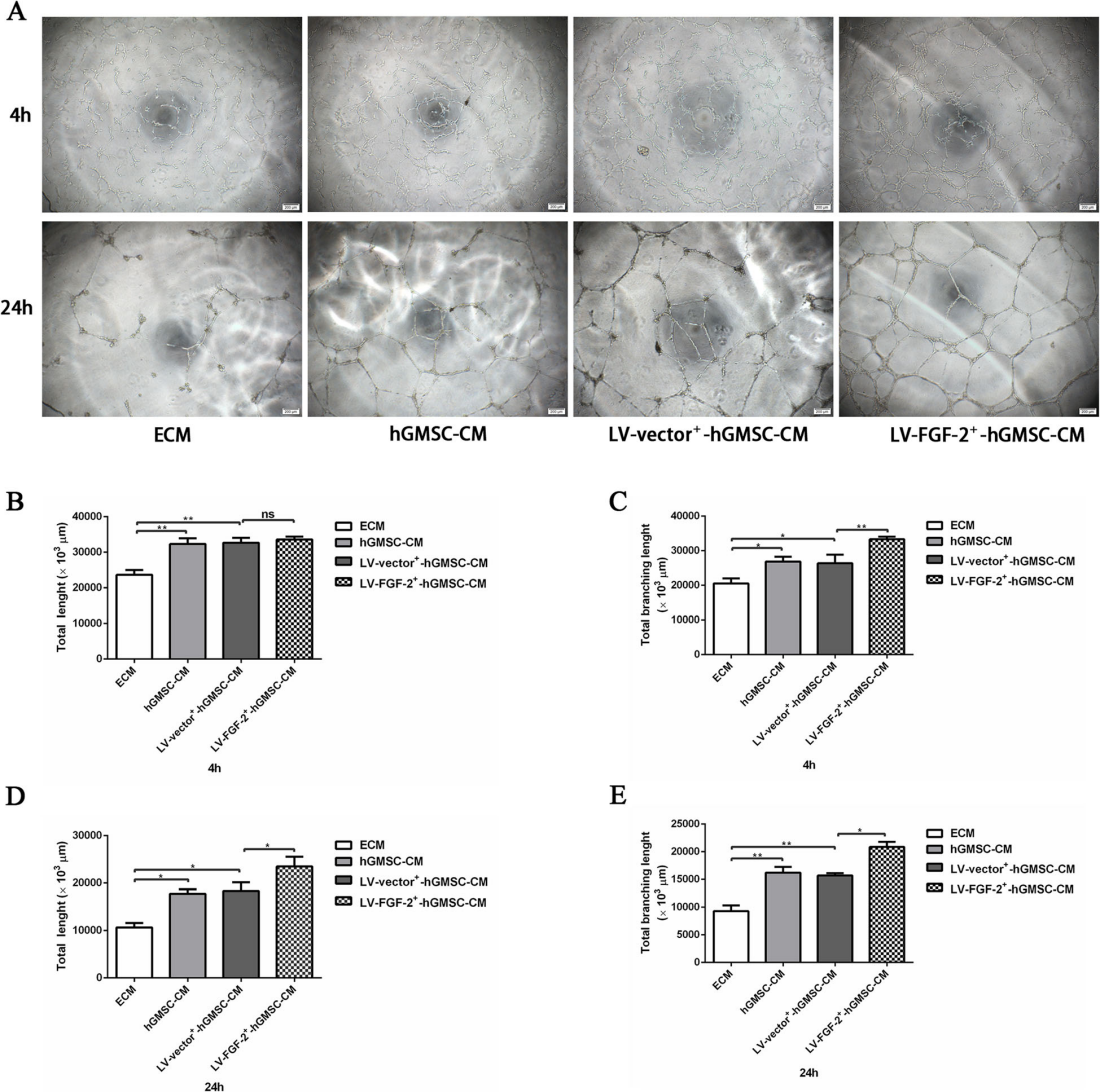
The effect of LV-FGF-2^+^-hGMSC-CM on the tube formation of HUVECs in vitro. HUVECs were cultured on the matrigel matrix with or without different CMs added. The tube-like structures were photographed under an inverted microscope at × 40 magnification and the total tube length and the total branching length analyzed by ImageJ software at 4 h (**a**–**c**) or 24 h (**a**, **d**, and **e**). ns = no significant difference, *p < 0.05, **p < 0.01

To investigate the pro-angiogenic effects of LV-FGF-2^+^-hGMSC-CM, a tube formation assay was performed to observe vascular network formation by HUVECs. The results showed that at 4 h, there was no significant difference in total tube length among hGMSC-CM group, LV-vector^+^-hGMSC-CM, and LV-FGF-2^+^-hGMSC-CM group; however, the total tube length in these three groups was significantly longer than that in the negative control group (Fig. 5a, b). The total branching length in the LV-FGF-2^+^-hGMSC-CM group was significantly longer than that in the hGMSC-CM group and LV-vector^+^-hGMSC-CM group, which was significantly longer than that in the negative control group (Fig. 5a, c). At 24 h, both total tube length (Fig. 5a, d) and total branching length (Fig. 5a, e) in the LV-FGF-2^+^-hGMSC-CM group were significantly longer than those in the LV-vector^+^-hGMSC-CM group and hGMSC-CM group, which were significantly longer than in the negative control group.

### LV-FGF-2^+^-hGMSC-CM promotes angiogenesis in vivo

**Fig. 6:**
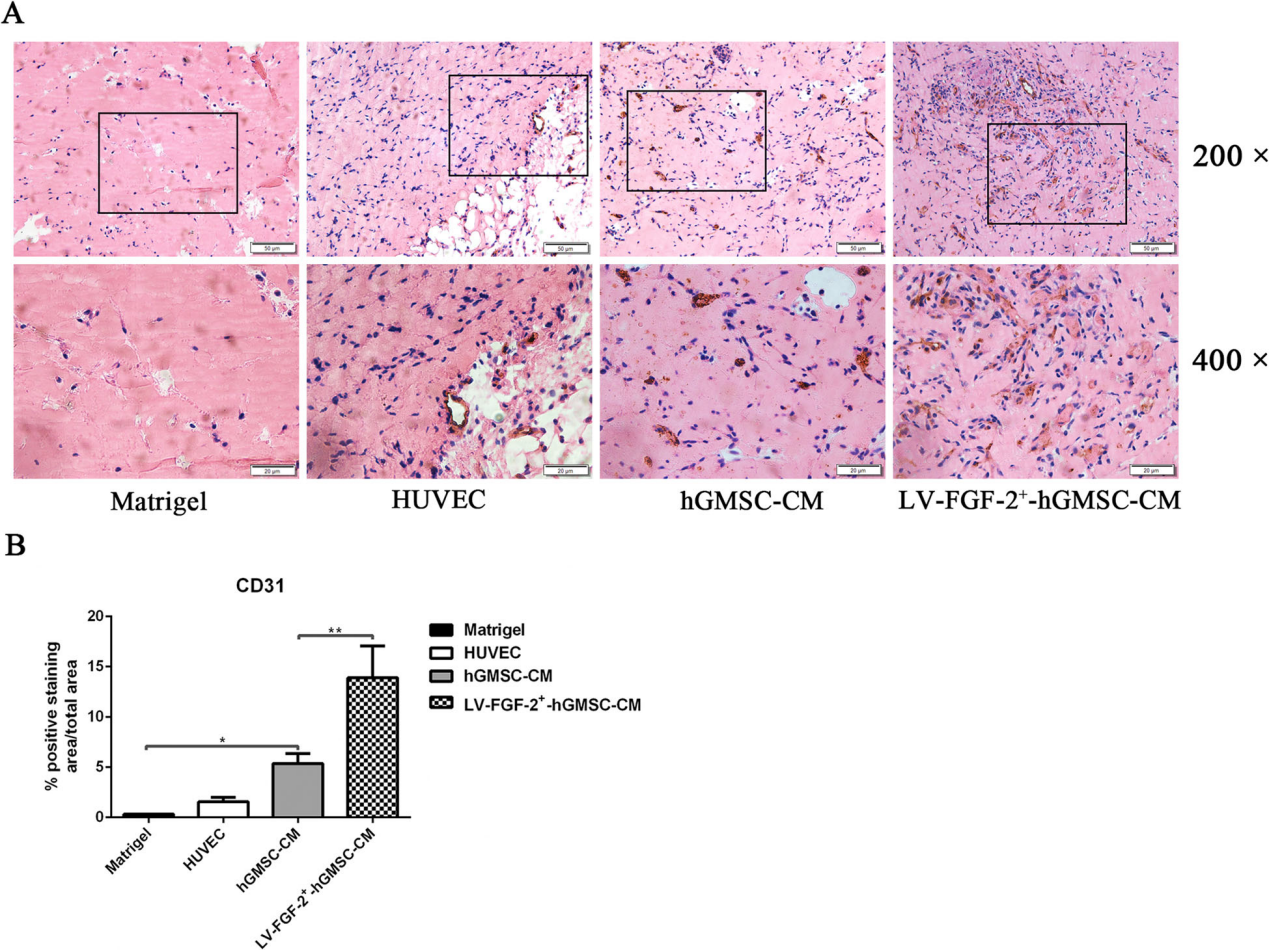
LV-FGF-2^+^-hGMSC-CM promotes angiogenesis in vivo. NOD-SCID mice were injected with matrigel, matrigel with HUVECs, matrigel with HUVECs and hGMSC-CM, or matrigel with HUVECs and LV-FGF-2^+^-hGMSC-CM. The plug sections were stained with anti-human CD31 antibody, and quantitative analysis was analyzed by using Image J software. *p < 0.05, **p < 0.01

To study the pro-angiogenesis ability of LV-FGF-2^+^-hGMSCs-CM in vivo, matrigel mixed with HUVECs, HUVECs with hGMSC-CM, or HUVECs with LV-FGF-2^+^-hGMSC-CM was injected subcutaneously into NOD-SCID mice and only matrigel was used as the control. After 7 days, blood vessels were analyzed by quantification of CD31 staining. As shown in Fig. 6, compared with plug enriched hGMSC-CM, HUVECs, or plug control, the plug-enriched LV-FGF-2^+^-hGMSC-CM showed a higher anti-CD31-positive capillary density (Fig. 6a, b). Although the anti-CD31-positive capillary density in plug-enriched hGMSCs was higher than that in the plug with HUVECs, there was no statistical difference between them (Fig. 6a, b). In summary, the conditioned medium derived from FGF-2 gene overexpressed hGMSCs had a stronger ability promoting angiogenesis than that derived from hGMSCs.

## Discussion

In this study, in order to optimize CM for the enhanced potential of angiogenesis, we used lentivirus as a vector to overexpress the FGF-2 gene into hGMSCs. Results demonstrated that FGF-2 gene transfection increased the expression and secretion of angiogenesis-related factors such as VEGF, FGF-2, and TGF-β in hGMSCs. LV-FGF-2^+^-hGMSC-CM had a stronger pro-angiogenesis and migration promotion effect on HUVECs than LV-vector^+^-hGMSC-CM.

Growth factor VEGF has been a hot spot for studying angiogenesis, and its role in angiogenesis and tissue repair or regeneration has attracted wide attention [23, 24, 33–36]. It can enhance both intra-articular ossification and intramembranous bone formation [34, 37], meanwhile can recruit stem cells to damaged or diseased bone tissue [33, 36]. However, studies have also shown that the use of VEGF alone does not promote the healing of bone damage and the combination with other growth factors has a stronger and more comprehensive role in promoting tissue healing [35]. TGF-β plays an important role in the physiological and pathological processes associated with tissue remodeling. In terms of angiogenesis, TGF-β upregulates the production of TSP-4, a pro-angiogenic extracellular matrix protein in cultured endothelial cells (EC) [38], thereby promoting angiogenesis. It also augments ECM protein expression via the SMAD signaling pathway to remodel ECM to promote blood vessel production [39]. Given the importance of VEGF and TGF-β in angiogenesis, elevating their concentration should promote angiogenesis potentials. In this study, FGF-2 gene transfection increased the secretion of VEGF and TGF-β in hGMSCs. Importantly, FGF-2 itself is an important inducer for angiogenesis. All these suggest that FGF-2-overexpressed hGMSCs may be a perspective medication tool for angiogenesis.

Successful bone defect healing requires simultaneous regeneration of both the mineralized tissue and vasculature to obtain the highly vascularized bone. During the vasculature formation process, a variety of mediators of angiogenesis induce migration and differentiation of endothelial progenitor cells (EPCs) to increase blood vessel density [40]. In this study, the effect of the CM obtained from FGF-2 overexpressed hGMSCs on HUVEC angiogenic ability was evaluated in vitro and in vivo. The results confirmed that hGMSC-CM enhanced gene and protein expressions of angiogenesis-related factors, tube-forming ability, and cell migration ability in HUVECs while LV-FGF-2^+^-hGMSC-CM had stronger efficacy compared with hGMSC-CM.

CD31, also known as platelet endothelial cell adhesionmolecule1 (PEMCAM1), is a differentiation marker of blood vessel EPCs [41]. It is one of the most famous immunohistochemical markers among vascular tumors, and its expression level is often used to reflect blood vessel density [42, 43]. In this study, we observed the expression level of CD31 in matrigel plug sections by immunohistochemistry. It was found that although hGMSC-CM promoted some blood vessel formation, the CM derived from FGF-2 gene-modified hGMSCs promoted more blood vessel formation.

Constitutive overexpression of FGF-2 in MSCs has a safety backlash. Just as pointed out by Fierro et al. [44], extensive safety and efficacy evaluation must be done before this type of cell/gene therapy could ever be considered. However, our proposed strategy is to promote the concentration of FGF2 and other pro-angiogenic factors in the CM, while the application of the CM is controllable. Recombinant human fibroblast growth factor (rhFGF)-2 has been used clinically in periodontal regeneration therapy [45], whereas FGF-2-modified MSC-derived CM provides some advantage over exogenous supplement of FGF-2 into the CM in that FGF-2-modified GMSCs not only produce and release more FGF-2, but also more VEGF-A, and TGF-β vs primary GMSCs. Briefly, an optimized conditioned medium by overexpressing the FGF-2 gene potentiates the angiogenic ability of hGMSCs.

## Conclusion

In summary, the present investigation reveals that CM from FGF-2-modified hGMSCs contains more VEGF-A, FGF-2, and TGF-β and has a stronger pro-angiogenic and migration promotion effect on HUVECs than on hGMSC-CM in vitro and in vivo. Nevertheless, many further tasks need to be conducted to evaluate the advantage of this strategy. For example, the deeper in vivo experiment is required to observe whether FGF-2-modified hGMSC-CM promotes a simultaneous regeneration of both the mineralized tissue and vasculature to obtain the highly vascularized bone.

## Supplementary information

**Additional file 1.** Plasmid map. The map of plasmid showed that the lentivirus vector pHBLV-CMV-MCS-3FLAG-EF1-ZsGreen-T2A-PURO-FGF-2 was successfully constructed.

## Data Availability

The datasets used and/or analyzed during the current study are included in this published article or available from the corresponding author on reasonable request.

## Abbreviations

BMMSCs: Bone marrow mesenchymal stem cells
CKO: Conditional knockout
CM: Conditioned medium
FGF-2: Fibroblast growth factor-2
hGMSCs: Human gingival MSCs
HUVECs: Human umbilical vein endothelial cells
LV-FGF-2^+^-hGMSCs: FGF-2 gene-modified hGMSCs were constructed by lentiviral transfection technology
MSC-CM: Conditioned medium derived from mesenchymal stem cells
MSCs: Mesenchymal stem cells
PDLSCs: Stem cells from periodontal ligaments
PLGF: Placenta growth factor
SCF: Stem cell factor
TGF-β: Transforming growth factor β
VEGF-A: Vascular endothelial growth factor A
VEGFR2: Vascular endothelial growth factor receptor 2
