# Supplementary figures and images for: Conditioned medium derived from FGF-2-modified GMSCs enhances migration and angiogenesis of human umbilical vein endothelial cells
Source: Stem Cell Res Ther. 2020 Feb 18;11:68. doi: 10.1186/s13287-020-1584-3 (PMC7029497; doi:10.1186/s13287-020-1584-3)

# Plasmid map

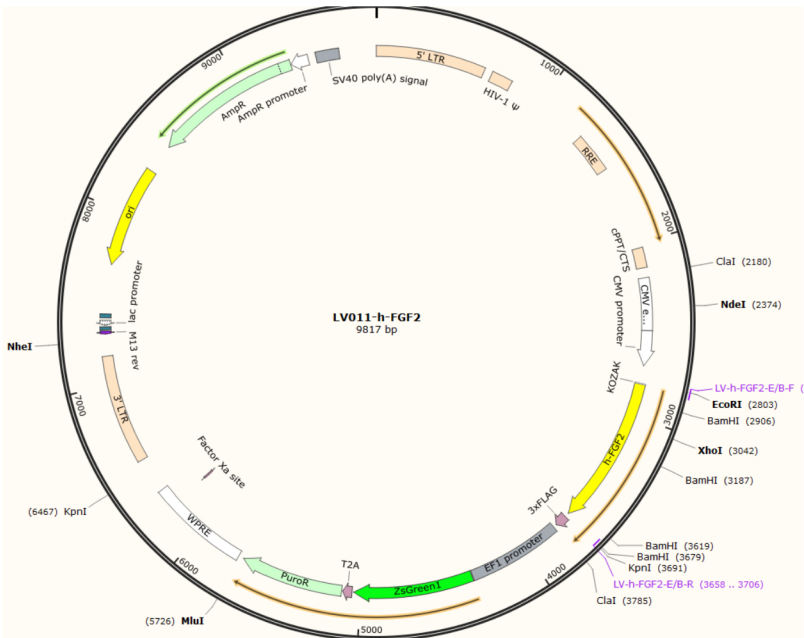

Supplement: Supplementary file 1 — Additional file 1. Plasmid map. The map of plasmid showed that the lentivirus vector pHBLV-CMV-MCS-3FLAG-EF1-ZsGreen-T2A-PURO-FGF-2 was successfully constructed. [file 13287_2020_1584_MOESM1_ESM.pdf]
